# Indole 3-Butyric Acid Metabolism and Transport in *Arabidopsis thaliana*

**DOI:** 10.3389/fpls.2019.00851

**Published:** 2019-07-03

**Authors:** Suresh Damodaran, Lucia C. Strader

**Affiliations:** ^1^Department of Biology, Washington University in St. Louis, St. Louis, MO, United States; ^2^Center for Science and Engineering Living Systems, Washington University in St. Louis, St. Louis, MO, United States; ^3^Center for Engineering MechanoBiology, Washington University in St. Louis, St. Louis, MO, United States

**Keywords:** auxin, indole-3-butyric acid, phytohormone, ATP-binding cassette transporter, TRANSPORTER OF IBA1, transporters

## Abstract

Auxin is a crucial phytohormone involved in multiple plant developmental processes. Spatiotemporal regulation of auxin levels is necessary to achieve development of organs in the proper place and at the proper time. These levels can be regulated by conversion of auxin [indole 3-acetic acid (IAA)] from its conjugated forms and its precursors. Indole 3-butyric acid (IBA) is an auxin precursor that is converted to IAA in a peroxisomal β-oxidation process. In Arabidopsis, altered IBA-to-IAA conversion leads to multiple plant defects, indicating that IBA contributes to auxin homeostasis in critical ways. Like IAA, IBA and its conjugates can be transported in plants, yet many IBA carriers still need to be identified. In this review, we discuss IBA transporters identified in Arabidopsis thus far, including the pleiotropic drug resistance (PDR) members of the G subfamily of ATP-binding cassette transporter (ABCG) family, the TRANSPORTER OF IBA1 (TOB1) member of the major facilitator superfamily (MFS) family and hypothesize other potential IBA carriers involved in plant development.

## Introduction

Multiple pathways coordinate plant development; many of which require auxin for their effect on development. Auxin is a well-studied plant hormone that is important for multiple plant developmental processes with major roles in cell division, differentiation, and elongation (Ljung, 2013; reviewed in Zhao, 2010). Endogenous active auxins include indole acetic acid (IAA), phenyl acetic acid (PAA), and 4-chloroindole-3-acetic acid (4-Cl-IAA) (Cook, 2019; reviewed in Korasick et al., 2013). IAA is widely considered to be the predominant form of active auxin within the plant and likely contributes to the majority of auxin activity in many plants.

Attaining auxin maxima in specific tissues is essential for organogenesis and for environmental responses. Mechanisms that contribute to the regulated distribution of auxin include polar auxin transport and IAA metabolism. The polarized localization of IAA transporters regulates auxin movement in specific directions, whereas local auxin metabolism provides the IAA for this transport and is also critical for establishing auxin maxima. These metabolism and transport mechanisms for IAA likely act in concert (Brumos et al., 2018; Morffy and Strader, 2018).

Auxin is primarily synthesized through a two-step tryptophan-dependent auxin biosynthesis pathway catalyzed by the tryptophan aminotransferase of Arabidopsis (TAA) and YUCCA (flavin monooxygenase enzyme) families of enzymes (reviewed in Zhao, 2012). TAA family enzymes convert the aromatic amino acid tryptophan into indole-3-pyruvic acid (IPyA); this is subsequently converted into IAA by YUCCA family members. Higher order *taa* and higher order *yucca* mutants display drastic developmental phenotypic defects (Zhao et al., 2001; Cheng et al., 2006, 2007; Stepanova et al., 2008; Tao et al., 2008), suggesting functional redundancy within these families and also revealing critical roles for *de novo* auxin biosynthesis in plant development.

In addition to *de novo* auxin biosynthesis, IAA can be released from conjugates with sugars, amino acids, and the chain-lengthened precursor indole-3-butyric acid (IBA) (reviewed in Korasick et al., 2013; Frick and Strader, 2018). Several amide-linked IAA conjugates have been identified in plants, including IAA-Ala and IAA-Leu, which can be hydrolyzed to release free IAA in Arabidopsis, maize, and several other plant species (Bartel and Fink, 1995; LeClere et al., 2002; Campanella et al., 2003a,b, 2008; Rampey et al., 2004). IAA stored in the form of an ester-linked IAA-sugar conjugate is hydrolyzed to release active IAA in both monocots and dicots (Jakubowska et al., 1993; Jakubowska and Kowalczyk, 2005; Campanella et al., 2008). The auxin precursor IBA is converted in to active IAA through a β-oxidation process in the peroxisome (Strader et al., 2011).

### Metabolism of Indole 3-Butyric Acid and Its Role as an Auxin Precursor

IBA was initially assumed to be a synthetic auxinic compound that was primarily used as a rooting media agent. In marigold, tomato, buckwheat, pea, bean, sunflower, and few other plant species, exogenous IBA induces root elongation, leaf epinasty, and stem bending (Zimmerman and Wilcoxon, 1935). Although IBA was once thought to be a synthetic auxin, it was later detected as an endogenous compound in potato peelings using paper chromatography (Blommaert, 1954). Endogenous IBA has been detected in multiple plant species, including Arabidopsis, tobacco, pea, and maize (reviewed in Korasick et al., 2013). This widespread occurrence of IBA suggests that this molecule may play a conserved role across species.

Although IBA has been identified as an endogenous compound in multiple plant species, it is often present at low levels and is difficult to detect. Indeed, the presence of IBA as an endogenous molecule has been questioned in one study, due to an inability to detect it using gas chromatography-mass spectrometry and liquid chromatography-mass spectrometry in Arabidopsis, poplar, and wheat (Novak et al., 2012). In studies in which endogenous IBA has been detected, its levels are often found at a lower level than IAA (Sutter and Cohen, 1992; Ludwig-Müller et al., 1993). Among these studies, differences in IBA detection could reflect (1) differences in metabolite extraction techniques, (2) detection limits of the technology being used, and/or (3) differences in IBA accumulation in plants grown under distinct laboratory conditions. IBA contribution to overall auxin level varies among species and even among accessions within a single species, suggesting differential IBA metabolism in different plants (Ludwig-Müller and Epstein, 1991; Ludwig-Müller et al., 1993). Resolving these differences in the future will be key to understanding the prevalence of IBA contributions to the auxin pool.

**Figure 1:**
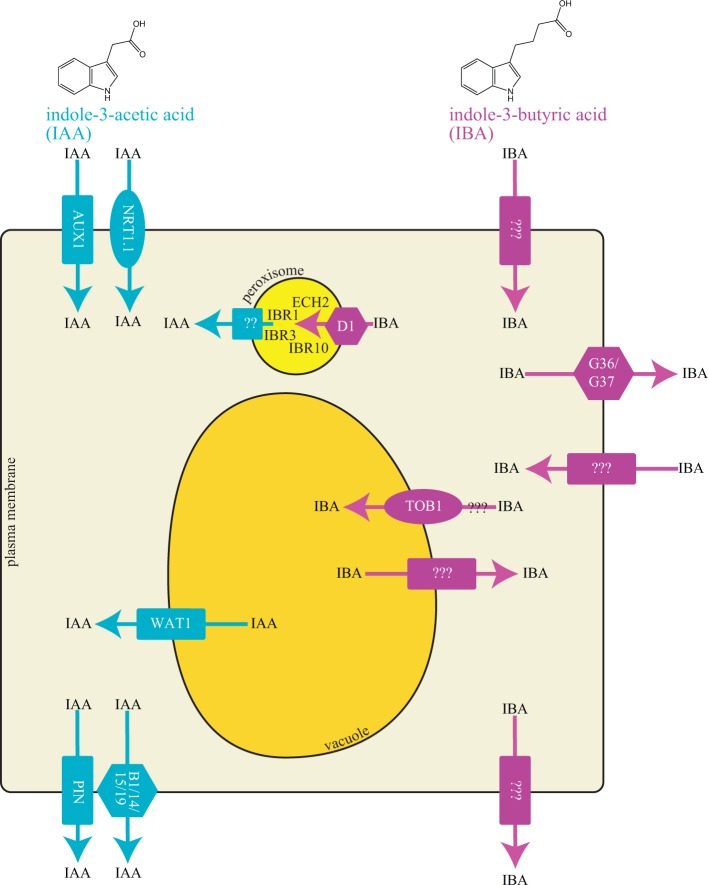
Cellular model of IAA and IBA transporters. IAA and IBA are indolic compounds with two and four carbon side chains, respectively. Graphical representation of a cell with different transport proteins involved in transport of IAA and IBA.

IBA is structurally similar to IAA except for the side chain, in which IAA has two carbons and IBA has a four-carbon side chain (Figure 1). IAA binds to the TIR1/AFB-Aux/IAA (TRANSPORT INHIBITOR RESPONSE 1/AUXIN SIGNALING F-BOX PROTEIN-Auxin/INDOLE-3-ACETIC ACID) co-receptor complex to initiate downstream auxin-responsive gene expression (reviewed in Lavy and Estelle, 2016). Because of its lengthened side chain, IBA is unable to stimulate formation of the auxin co-receptor complex (Uzunova et al., 2016). Thus, physiological effects of IBA treatment are likely caused by IBA-derived IAA and not by the IBA molecule itself.

The major mechanism by which IBA influences plant development is through the reduction of its carbon side chain to convert in to IAA (reviewed in Strader and Bartel, 2011). IBA-to-IAA conversion occurs through a fatty acid β-oxidation process housed in the peroxisome (Zolman et al., 2000; Strader et al., 2010). Enzymes specifically involved in catalyzing IBA conversion include the short-chain dehydrogenase/reductase indole-3-butyric acid response 1 (IBR1; Zolman et al., 2007), the acyl-coA dehydrogenase/oxidase-like IBR3 (Zolman et al., 2007), the predicted enoyl coA hydratase IBR10 (Zolman et al., 2008), and enoyl-COA hydratase2 (ECH2; Strader et al., 2011). In addition, generalist enzymes such as PED1 (3-ketoacyl COA thiolase) (Zolman et al., 2000) and ACX enzymes (Adham et al., 2005) may also act in the conversion of IBA to IAA. Peroxisomal fatty acid β-oxidation of oil bodies is required in Arabidopsis to fuel growth prior to photosynthesis. Thus, dark-grown seedlings defective in fatty acid β-oxidation display reduced growth unless the media is supplemented with an exogenous carbon source such as sucrose (Zolman et al., 2000). Because mutants defective in *IBR1*, *IBR3*, *IBR10*, or *ECH2* grow normally in the dark in the absence of sucrose for growth (Zolman et al., 2007, 2008; Strader et al., 2011), these enzymes are unlikely to be involved in fatty acid β-oxidation; however, it is possible they are involved in β-oxidation of substrates in addition to IBA. Conversely, mutants defective in PED1 or ACX enzymes are resistant to IBA and also require an exogenous carbon source to fuel dark-grown growth (Zolman et al., 2000; Adham et al., 2005), suggesting that these are in involved in both IBA and fatty acid β-oxidation.

Mutants defective in IBA-to-IAA conversion enzymes display multiple plant developmental defects, including reduced cotyledon expansion, reduced apical hook curvature, reduced lateral root formation, and smaller root apical meristems, along with decreased levels of free IAA (Zolman et al., 2008; Strader et al., 2010, 2011). IBA treatment fails to stimulate lateral root organogenesis in mutants defective in IBA conversion enzymes (Strader et al., 2011), suggesting that IBA cannot function directly to stimulate lateral root production but rather acts through its conversion to IAA (Strader et al., 2011). Further, the chemical naxillin stimulates lateral root production through stimulation of IBA-to-IAA conversion in Arabidopsis (De Rybel et al., 2012), confirming strong roles for IBA contribution to the pool of active auxin to regulate production of lateral roots.

Similar to IAA conjugates, both amide- and ester-linked conjugates of IBA have been detected in plants (Ludwig-Müller et al., 1993; Tognetti et al., 2010; Liu et al., 2012; Sherp et al., 2018). Overexpression of *UGT74E2*, encoding a glucosyltransferase, results in reduced IBA levels and elevated IBA-glucose conjugate levels in Arabidopsis (Tognetti et al., 2010), suggesting that UGT74E2 catalyzes conjugation of IBA to glucose. Further, Arabidopsis plants overexpressing *UGT74E2* display increased shoot branching and improved abiotic stress tolerance (Tognetti et al., 2010), consistent with the possibility that altered IBA homeostasis affects plant growth and stress response. In these experiments, it is unclear whether depletion of the IBA molecule itself or depletion of IBA-derived IAA affects growth and stress responses when *UGT74E2* is overexpressed. In addition to conjugation to glucose, IBA is also conjugated to amino acids (reviewed in Korasick et al., 2013). The Arabidopsis GH3-15 acyl acid amido synthetase enzyme specifically conjugates IBA to aspartate; however, whether this conjugated form is for IBA storage or for degradation is unknown (Sherp et al., 2018). Overall, these data suggest that homeostasis of IBA and its conjugates play important roles in maintaining IAA levels.

### Indole 3-Butyric Acid Transport

Polar auxin transport regulates IAA distribution *via* specific cellular carriers. Similar to IAA, IBA is moved by transporters (reviewed in Strader and Bartel, 2011; Michniewicz et al., 2014). Because IBA is structurally similar to IAA (Figure 1), the question arises whether IAA transporters can also transport IBA. IAA is transported directionally with the aid of tissue-specific influx and efflux proteins (reviewed in Vieten et al., 2007; Peer et al., 2011). Transporters involved in polar IAA transport include both uptake and efflux carriers. IAA uptake is carried out by members of the amino acid permease-like AUXIN RESISTANT1 (AUX1) family. Two transporter families mediate cellular IAA efflux: the family of PIN-FORMED (PIN) proteins and the MULTIDRUG RESISTANCE/GLYCOPROTEIN (PGP) class of ATP-binding cassette (ABC) transporters (reviewed in Vieten et al., 2007; Petrášek and Friml, 2009). No examined IAA transporter appears to mediate IBA transport (see sections below) suggesting the presence of IBA specific transporters in Arabidopsis.

Long-distance IBA transport was first suggested based on evidence that localized IBA treatment affected distal developmental events in marigold, tomato, sweet pea, and few other species (Zimmerman and Wilcoxon, 1935). Long-distance transport of radiolabeled IBA allowed tracking of IBA movement in cleopatra mandarin (Epstein and Sagee, 1992) and Arabidopsis (Figure 1; Ludwig-Müller et al., 1995; Rashotte et al., 2003). However, a major caveat to these tracking experiments is that the identity of the tracked molecule is unknown; thus, movement of [^3^H]IBA is indistinguishable from movement of [^3^H]IBA-conjugates or [^3^H]IAA derived from [^3^H]IBA. To overcome this limitation, analytical methods have been used to determine the identity of IBA-derived molecules in transport assays (Ruzicka et al., 2010; Liu et al., 2012). Application of [^3^H]-IBA to Arabidopsis root columella cells results in high-performance liquid chromatography-based detection of [^3^H]-IAA in root tissue 4 mm above the application site after a 2-h incubation (Ruzicka et al., 2010), suggesting that most of the transported molecule in this assay was IBA-derived IAA. Further, gas chromatography-mass spectrometry (GC/MS) methods were used to determine transported molecules across the Arabidopsis hypocotyl and inflorescence stem after heavy IBA application; multiple IBA-derived molecules were transported through these tissues, including IAA and ester-linked IBA (Liu et al., 2012). By comparison, movement of heavy IBA through these tissues occurred at lower levels, suggesting that, in Arabidopsis, IBA metabolites are transported long distances more efficiently than the IBA molecule itself (Liu et al., 2012). Thus, it seems that much of the long-distance transport of “IBA” may be of IBA conjugates, rather than the IBA molecule itself. Further, no identified IBA transporter has been shown to have roles in long-distance IBA transport.

IBA uptake is a saturable process (Ludwig-Müller et al., 1995; Rashotte et al., 2003), suggesting that IBA uptake is mediated by carriers rather than by simple diffusion. IBA transport is unaltered in the *aux1* mutant in both long-distance (Rashotte et al., 2003) and root tip (Strader and Bartel, 2009) transport assays. Thus, IBA is likely not a substrate for AUX1; however, it remains possible that it may be a substrate for other members of the AUX1 family. In particular, LIKE AUX1 (LAX3) appears to display some affinity toward IBA when heterologously expressed in *Xenopus* oocytes (Swarup et al., 2008), suggesting that this transporter may use IBA, in addition to IAA, as a substrate.

IBA efflux appears to be mediated by carriers distinct from IAA efflux carriers. Application of the polar auxin transport inhibitors 1-N-napthylphthalamic acid (NPA) or 2,3,5-triiodobenzoic acid (TIBA) fail to block IBA efflux, whereas they have a dramatic effect on IAA transport (Rashotte et al., 2003; Liu et al., 2012), suggesting independent efflux mechanisms for these two molecules. Further, heterologously expressed PIN2, PIN7, ABCB1, and ABCB19 display no IBA efflux activity (Ruzicka et al., 2010). Thus, plants seem to use distinct efflux carriers for IBA and IAA. Indeed, several transporters that use IBA, but not IAA, as a substrate have been described. Using genetic and molecular approaches in Arabidopsis, IBA transporters including PXA1/ABCD1, ABCG36, ABCG37, and TOB1 have been identified, and their roles are elaborated in the following text.

#### Peroxisomal Indole 3-Butyric Acid Transporter-PXA1

IBA to IAA conversion occurs in the peroxisome; thus, an intracellular carrier is necessary for movement of IBA into peroxisomes. The peroxisomal ABC transporter PEROXISOMAL ABC TRANSPORTER1/ABCD1/PXA1 appears to be the influx carrier involved in transporting IBA for IAA conversion through the β-oxidation process (Figure 1; Zolman et al., 2001b). The loss-of-function *pxa1* mutant displays resistance to the long-chain auxin precursor IBA, but wild-type sensitivity to the active auxin IAA (Zolman et al., 2001b), due to a reduction in IBA-to-IAA conversion (Strader et al., 2010). PXA1 likely transports compounds in addition to IBA for peroxisomal β-oxidation, such as fatty acid and jasmonic acid (Zolman et al., 2001a; Linka et al., 2008; Kunz et al., 2009). The peroxisomal transporter required for efflux of IBA-derived IAA from the peroxisome is unknown.

#### Pleiotropic Drug Resistance Proteins Functions as Indole 3-Butyric Acid Efflux Transporters

The ABCG36 and ABCG37 members of the pleiotropic drug resistance (PDR) subclade of the ABCG family of ATP-binding cassette transporters (Strader et al., 2008; Strader and Bartel, 2009; Ruzicka et al., 2010) are required for efflux of IBA from the root. Mutants defective in ABCG36 were found in a screen for altered IBA sensitivity (Strader and Bartel, 2009). Mutants defective in ABCG37 were identified in screens for altered sensitivity to 2,4-dichlorophenoxyacetic acid (2,4-D) (Ito and Gray, 2006), for altered sensitivity to polar auxin transport inhibitors (Ruzicka et al., 2010), or for altered sensitivity to IBA (Strader et al., 2008). Excised root tips from *abcg36* and *abcg37* loss-of-function mutants hyperaccumulate [^3^H]-IBA, but not [^3^H]-IAA, suggesting that the ABCG36 and ABCG37 transporters act to efflux IBA (Figure 1; Strader et al., 2008; Strader and Bartel, 2009; Ruzicka et al., 2010). The hyperaccumulation of [^3^H]-IBA, combined with the localization of ABCG36 and ABCG37 to the outer polar domain of root epidermal and lateral root cap cells (Stein et al., 2006; Strader and Bartel, 2009; Ruzicka et al., 2010), suggest that these transporters move IBA out of the root.

To determine whether ABCG37 directly transports IBA, it was expressed in *Schizosaccharomyces pombe* (see Table 1), in which ABCG37-expressing cells accumulated less [^3^H]-IBA compared to control cells, with no change in [^3^H]-IAA accumulation (Ruzicka et al., 2010), suggesting that ABCG37 directly transports IBA. Although ABCG36 has not been evaluated in a heterologous system for efflux activity, the IBA hyperaccumulation in the *abcg36* mutant (Strader and Bartel, 2009) suggests that ABCG36 could be functionally similar to ABCG37 in IBA transport. Further, the *abcg36 abcg37* double mutant displays additive IBA hypersensitivity and [^3^H]-IBA hyperaccumulation in comparison to either the *abcg36* or *abcg37* single mutants, suggesting overlapping functions (Ruzicka et al., 2010).

**Table 1:** Heterologous expression systems for transport assays.

Heterologous system	Pros and cons	Auxin-related transporters characterized
Yeast*–Saccharomyces cervisiae* and *Schizosaccharomyces pombe*	Easy to manipulate and cultivate in standard lab conditions. Allows proper folding and translocation of transmembrane proteins. Overexpression can lead to aggregates of misfolded protein.	ABCB, AUX, and PIN family proteins (Yang and Murphy, 2009). TOB1 (Michniewicz et al., 2019).
*Xenopus* oocyte–Oocytes harvested from the South African clawed frog *Xenopus laevis*	Electrophysiological measurements using the two-electrode voltage clamp technique. Large oocyte size facilitates handling and microinjection. Exogenous transporters post-translationally modified before plasma membrane localization.	PIN (Zourelidou et al., 2014), TOB1 (Michniewicz et al., 2019).
Insect cell–Baculovirus/*Spodoptera frugiperda* (Sf9) insect cells	Ability to express large quantities of eukaryotic transmembrane protein. Media is expensive and protein yield is minimal. Need to infect cells with virus for protein expression each time; stable lines difficult to maintain. Tissue culture conditions needed.	AUX1 (Carrier et al., 2008), ABCG25 (Kuromori et al., 2010)
Mammalian cell lines such as HeLa	Standardized protocols available for transfection and stable expression of transmembrane protein Media is expensive and protein yield is minimal. Tissue culture conditions needed.	ABCG37 (Ruzicka et al., 2010)
Plant protoplast tobacco BY-2 cells	Transformation protocols are standardized. Environment similar to native environment of transporter. Possible interference by endogenous transmembrane proteins. Unclear whether transport activity is due to direct transport or by modulation of existing plant transporters.	

ABCG37 and ABCG36 appear to transport substrates in addition to IBA. PDR proteins typically have broad range of substrates (reviewed in van den Brule and Smart, 2002; Verrier et al., 2008). ABCG37 displays broad substrate specificity, including several auxinic compounds such as the synthetic auxin 2,4-D (Ito and Gray, 2006; Strader et al., 2008), 2,4-dichlorophenoxy butyric acid (2,4-DB) (Strader et al., 2008; Ruzicka et al., 2010), and the auxin transport inhibitor (NPA) (Ito and Gray, 2006). ABCG36 likely transports cadmium (Kim et al., 2007) and the indolic defense compound 4-methoxyindol-3-yl-methanol (Matern et al., 2019).

ABCG36 and ABCG37 play roles in distinct areas of plant development. In particular, the *abcg36* mutant displays an increased number of lateral roots, larger cotyledons, and increased root hair lengths (Strader and Bartel, 2009), consistent with elevated auxin levels in these tissues. ABCG36 and ABCG37 localize to the outward face of epidermal cells in leaves (ABCG36) and roots (ABCG36 and 37) (Strader and Bartel, 2009; Langowski et al., 2010; Ruzicka et al., 2010), suggesting that these transporters exude IBA into the environment. This activity may be crucial for maintaining IBA levels and auxin homeostasis, as *abcg36* and *abcg37* mutant display phenotypes consistent with elevated auxin accumulation. Further, blocking IBA-to-IAA conversion in the *abcg36* mutant suppresses these phenotypes (Strader et al., 2011), suggesting that IBA conversion is necessary for the developmental defects in *abcg36*. In roots, ABCG36 and ABCG37 are polarly localized to the outward-facing plasma membrane of root epidermal and lateral root cap cells (Strader and Bartel, 2009; Langowski et al., 2010; Ruzicka et al., 2010) and may exude IBA into the rhizosphere, which could have potential implications on the microbial diversity.

#### TRANSPORTER OF IBA1 Transports Indole 3-Butyric Acid

TRANSPORTER OF IBA1 (TOB1) was identified in a forward genetics screen for suppression of *abcg36* IBA hypersensitivity (Michniewicz et al., 2019). TOB1 belongs to the NTR1 PTR FAMILY (NPF), a part of the larger group of major facilitator superfamily (MFS) transporters (Leran et al., 2014). The *tob1* loss-of-function mutant displays mild resistance to 2,4-DB and IBA and wild-type sensitivity to IAA and 2,4-D in root elongation assays (Michniewicz et al., 2019). Indeed, transport assays using excised Arabidopsis root tips reveal reduced accumulation of [^3^H]-IBA and no difference in [^3^H]-IAA accumulation in *tob1* compared to wild type root tips (Michniewicz et al., 2019). Further, TOB1 directly transports IBA in heterologous systems (see Table 1) such as yeast and *Xenopus* oocytes (Michniewicz et al., 2019). In addition, TOB1 transports nitrate (He et al., 2017; Michniewicz et al., 2019). In oocytes, nitrate serves as a better substrate than IBA, although IBA can compete with nitrate uptake in *TOB1*-expressing oocytes (Michniewicz et al., 2019).

TOB1 localizes to the vacuolar membrane and is primarily expressed in cells adjacent to the lateral root primordia and in the lateral root cap cells of the primary root (Michniewicz et al., 2019). The *tob1* loss-of-function mutants display increased numbers of lateral roots and altered root system architecture (Michniewicz et al., 2019), suggesting that TOB1 normally limits production of lateral roots, possibly by sequestering IBA to the vacuole to limit its contributions to the pool of active auxin in these tissues. *TOB1* is a direct target of the cytokinin reponse regulator ARR10 (Zubo et al., 2017) and *TOB1* expression is induced by cytokinin treatment (Michniewicz et al., 2019). Further, the *tob1* mutant is resistant to the inhibitory effects of cytokinin on lateral root production (Michniewicz et al., 2019), suggesting that cytokinin’s inhibitory effects on lateral root production are at least partially mediated by moving IBA into the vacuole. These data suggest a model in which cytokinin regulates *TOB1* expression to regulate IBA contributions to auxin homeostasis during lateral root development.

Thus far, four proteins, PXA1 (Zolman et al., 2001b), ABCG36 (Strader and Bartel, 2009), ABCG37 (Strader et al., 2008; Ruzicka et al., 2010), and TOB1 (Michniewicz et al., 2019) have been implicated in IBA transport. Identification of these transporters has demonstrated the significance of IBA transporters in regulating aspects of development. Although progress has been made in identification of IBA transporters and IBA metabolic enzymes, much remains to be uncovered. Identifying additional transporters and determining their substrates (either IBA or IBA conjugates), combined with a detailed knowledge of IBA metabolism, will shine more light into understanding the plant developmental pathways.

Remaining questions on IBA transport:

- *How and where is IBA made and metabolized?* IBA may be derived from IAA in maize (Ludwig-Müller and Epstein, 1991; Ludwig-Müller et al., 1995a) and Arabidopsis (Ludwig-Muller, 2007). Yet, enzymes involved in IBA biosynthesis remain unidentified. IBA-derived auxin appears to be important in very specific aspects of plant development; it seems an inefficient system to create IBA from IAA, just to metabolize it to IAA again. Because IBA is important for early seedling growth, a stage in which peroxisomal activity is high in metabolizing storage oils, and because IBA β-oxidation releases not only free IAA but also acetyl-CoA, it may be possible that IBA is used in this scenario to not only provide auxin, but also energy, to drive growth. In addition, much of the transported IBA is in the form of IBA ester-linked conjugates (likely conjugated to sugars) (Liu et al., 2012). Whether IBA conjugates are made in one tissue; then transported to another for metabolism remains an open question. A greater understanding of tissue-type expression of IBA conversion enzymes and IBA transporters will be beneficial in understanding this system.
- *Roles for additional ABCG or TOB proteins in IBA transport.* Known IBA transporters belong to the PDR clade of the ATP-binding cassette (ABC) family and of the NPF clade of the major facilitator superfamily. Additional members of these clades may be involved in mediating IBA transport. In particular, mutants defective in ABCG29/PDR1 and ABCG33/PDR5 display hypersensitivity to the synthetic IBA mimic 2,4-DB (Michniewicz et al., 2014), consistent with the possibility that, similar to ABCG36 and ABCG37 family members, ABCG29 and ABCG33 transport IBA. In addition, TOB1 also has six closely related paralogs, which could function, similar to TOB1, in transporting IBA (Michniewicz et al., 2019). Functional characterization of these close paralogs could reveal potential IBA carriers. To determine whether these are *bona fide* IBA transporters, IBA transport activity must be demonstrated in a heterologous system (Table 1) such as yeast, *Xenopus* oocytes, insect cells, or mammalian cells (Haferkamp and Linka, 2012). There is a possibility that TOB1 paralogs (Michniewicz et al., 2019) could transport IBA in distinct tissues of Arabidopsis and promote the development of different tissues. Characterization of these members in addition to other potential transporters would aid in understanding the role of IBA transporters function at spatial levels to indirectly regulate IAA levels.
- *IBA uptake carriers.* IBA uptake is a rate-limited process (Rashotte et al., 2003), suggesting that its import into the cell is mediated by a carrier, rather than the effect of diffusion. Thus far, no IBA uptake carriers have been reported. A potential candidate for an uptake carrier is RESISTANT TO IBA1 (RIB1). The semi-dominant mutant *rib1* displays IBA resistance (Poupart and Waddell, 2000) and altered IBA transport (Poupart et al., 2005), consistent with the possibility that RIB1 mediates IBA uptake. Identification of the underlying mutation and molecular characterization of the gene product will allow for understanding the role of RIB1 in IBA transport.
- *Directionality of TOB1 transport*. The reduced 3H-IBA accumulation in *tob1* mutants combined with developmental phenotypes consistent with increased IBA contributions to the auxin pool suggests that TOB1 sequesters IBA in to vacuole (Michniewicz et al., 2019). Conversely, *tob1* resistance to the inhibitory effects of exogenous IBA on root elongation and electrophysiology assays are consistent with the possibility that TOB1 moves IBA out of the vacuole (Michniewicz et al., 2019). These contradictory pieces of data may reflect concentration-dependent differences in TOB1 transport direction. Thus, the direction of IBA movement by TOB1 remains unclear (Figure 1).
- *Identity of transported molecules.* Although it is clear that PDR9 and TOB1 directly transport IBA based on heterologous transport assays, it is not clear whether IBA itself or IBA-derived molecules are being tracked in other assays. Liu et al. (2012) elegantly used heavy IBA and mass spec analysis to determine that, in Arabidopsis hypocotyl tissues, most of the transported material was IBA-derived IAA or IBA conjugates. Likewise, in Ruzicka et al. (2010), [^3^H]-IBA applied to the columella was IBA-derived [^3^H]-IAA when sampled 4 mm from the root tip. Clearly, analytical methods need to be combined with transport assays for both IBA and IAA. Indeed, transport assays in heterologous systems, such as *Xenopus* oocytes, can be done on a time scale for which metabolism of molecules is not a factor.
- *IAA efflux carrier in peroxisome*. IBA-to-IAA conversion occurs in the peroxisome (reviewed in Figure 1; Strader and Bartel, 2011). However, the transporter involved in movement of IBA-derived IAA from the peroxisome into the cytoplasm is unidentified.
- *Long distance IBA efflux carriers.* Although long-distance transport has been speculated to exist based on radiotracer experiments, it seems that much of this tracked material was IBA metabolites, rather than free IBA. Certainly, none of the identified IBA transporters are involved in long-distance IBA transport. At this point, transporters involved in the long-distance transport of IBA or IBA conjugates remain elusive. Further, developmental roles for transport of IBA or IBA remain unknown; thus, it is possible that this mechanism is not a contributor to physiologically relevant processes.

## Author Contributions

All authors listed have made a substantial, direct and intellectual contribution to the work, and approved it for publication.

### Conflict of Interest Statement

The authors declare that the research was conducted in the absence of any commercial or financial relationships that could be construed as a potential conflict of interest.
